# Piezoelectric Energy Harvesting from the Thorax Vibration of Freely Flying Bees

**DOI:** 10.34133/cbsystems.0210

**Published:** 2025-02-26

**Authors:** Zhiyun Ma, Jieliang Zhao, Li Yu, Lulu Liang, Zhong Liu, Yongxia Gu, Jianing Wu, Wenzhong Wang, Shaoze Yan

**Affiliations:** ^1^ School of Mechanical Engineering, Beijing Institute of Technology, Beijing 100081, P. R. China.; ^2^ School of Artificial Intelligence, Beijing Technology and Business University, Beijing 100048, P. R. China.; ^3^ Department of Advanced Manufacturing, Sun Yat-sen University, Shenzhen 518107, P. R. China.; ^4^ Department of Mechanical Engineering, Tsinghua University, Beijing 100084, P. R. China.

## Abstract

Insect cyborgs have been proposed for application in future rescue operations, environmental monitoring, and hazardous area surveys. An energy harvester for insect carrying is critical to the long-lasting life of insect cyborgs, and designing an energy harvester with superior energy output within the load capacity of tiny flying insects is very important. In this study, we measured the thorax vibration frequency of bees during loaded flight conditions. We propose a piezoelectric vibration energy harvester for bees that has a mass of only 46 mg and can achieve maximum effective output voltage and energy density of 5.66 V and 1.27 mW/cm^3^, respectively. The harvester has no marked effect on the bees’ normal movement, which is verified by experiments of mounting the harvester on bees. These results indicate that the proposed harvester is expected to realize a self-power supply of tiny insect cyborgs.

## Introduction

Insect cyborgs (ICBs) represent a new generation of microaerial vehicles that can be controlled effectively by building an interface between biological and artificial systems [1]. Due to their superior aerodynamic performance and lower energy demand compared to those of conventional bionic aircraft [2], ICBs have been widely applied in urban and wilderness rescue operations [3], environmental monitoring [4], and hazardous area surveys [5,6]. Energy supply for microaerial vehicles has been challenging, especially for ICBs [7,8]. Currently, the research on ICBs has been focused on direct and indirect electrical stimulations of a motor system [9–13], where the power is provided by small batteries in control packs carried by insects [11,12]. Nevertheless, the size and weight of batteries are very large, accounting for up to 80% of the total device volume [14,15]. The frequent replacement or recharging of batteries severely undermines the disposable operational lifespan of ICBs and even causes damage to the vitality of the biocarriers [16].

With the continuous maturity of energy harvesting technology [17], related studies have been proposed to convert bio-vibration energy [18–21], bio-thermal energy [22–24], bio-energy [25–27], and surrounding environmental energy [28–30] into electrical energy to provide enough power for low-power electronic devices. The vibrational energy in organisms remains continuous and independent of the environment [31]. As miniaturization processing technology has matured, numerous researchers have focused on designing bio-vibrational energy harvesters for insects like moths [32], beetles [20], and bees [33]. However, due to bees’ low load capacity and high-frequency wing vibrations, creating lightweight, high-output energy harvesters with minimal disruption to their normal flight behavior is challenging [34]. Unfortunately, current studies lack a comprehensive consideration of the harvester’s mass and center of gravity when applied to freely flying bees [33]. Consequently, this leads to subpar energy harvesting device performance and restricts the bees’ freedom to fly, appreciably impacting their lifespan.

In this study, a frequency interval match-based method is proposed to harvest the vibration energy of bees in flight. First, an experimental analysis of the frequency of bee wing flapping was conducted under different load mass conditions. A piezoelectric energy harvester (PEH) that can effectively harvest the vibration energy of bees during flight was designed. The PEH performance was evaluated by Multiphysics field simulation methods and vibration experiments. Finally, the PEH was tested on living bees, and they were kept flying. The experimental results showed that the bees were well adapted to our PEH. This study can provide a guideline for the design of energy harvesters in bees and other flying insects.

## Materials and Methods

### Animal husbandry

The data of this study dealt with adult foraging worker bees (*Apis mellifera* L.). The bees were bought from Fragrant Hills Park in Beijing, China (40°N, 116°E), and were raised in an indoor glass beehive. The beehive was kept in a room temperature of 25 °C and a humidity of 50%. No specific permissions were required for these locations or activities. Moreover, the field studies did not involve any endangered or protected species.

### Bees’ wing-flapping frequency tests

A bee’s flight is powered by 2 muscles in the thorax, which occupy the majority of the thorax and drive the wingbeats through alternating high-frequency vibratory contractions, thus allowing the bee to complete the flight [35,36]. It has also been demonstrated that the vibration frequency of the bees’ thorax remains the same as the wing-flapping frequency under general flight conditions [37].

**Fig. 1.**
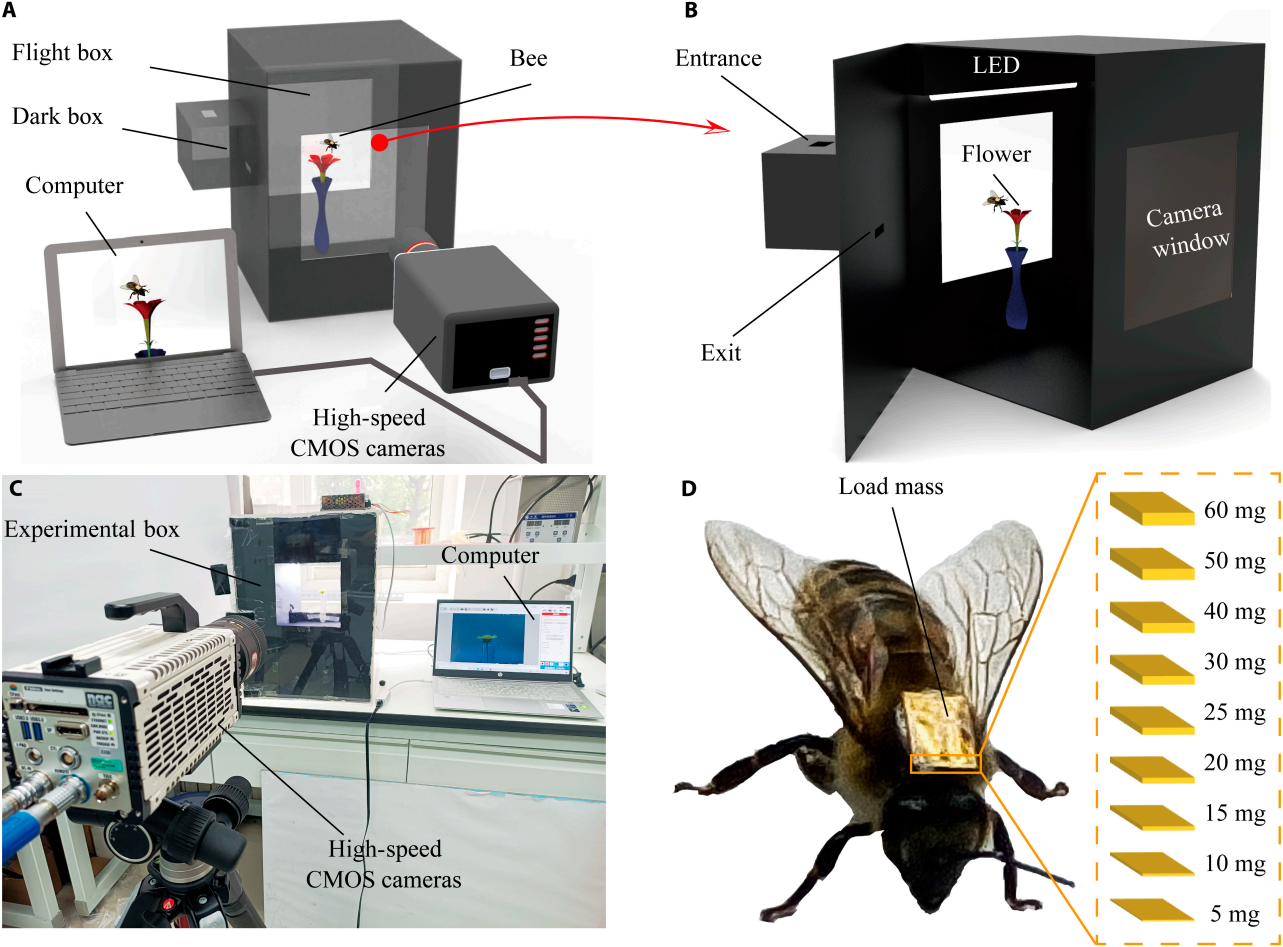
Experiment of bees flying with load masses. (A) Schematic diagram of the overall experimental setup. (B) The bees’ flight experiment box. (C) Photos of the experimental setup. (D) Schematic of the bees’ loading method and masses. CMOS, complementary metal–oxide–semiconductor; LED, light-emitting diode.

Figure 1A illustrates the experimental setup, where a loaded bee was placed inside an enclosure comprising a dark box and a flight box. A high-speed complementary metal–oxide–semiconductor (CMOS) camera recorded the bee’s flight. The dark box had an entrance and exit leading to the flight box. The flight box featured transparent windows on its front and back, serving both as shooting areas for the high-speed CMOS cameras and as entry points for natural light. To facilitate effective flight in the camera’s recording area, flowers of colors that strongly attracted bees (in this study, yellow) were placed inside the box. Then, the focus of the high-speed CMOS camera was adjusted on the flower. Additionally, light-emitting diode light strips were installed above the recording area to induce the flight behavior of bees, as shown in Fig. 1B. The experimental setup is shown in Fig. 1C; the bee sample used in this experiment was *A. mellifera.* In the experiments, bees were first frozen to reduce activity, and then adhesive was used to bond prepared mass blocks to their thorax. Once the bees had regained activity, they were placed in a dark box for 30 min. After that, the exit of the dark box was opened, allowing the bees to enter the flight box. The dark box was used to create a light-intensity gap, enhancing the bees’ flight effectiveness under light induction in the flight box. Once the bee entered the flight box and flew into the shooting area, the high-speed CMOS camera was quickly triggered to record the flight fragments. Then, we could obtain the wing-flapping frequency of bees by analyzing the frame rate of the camera. As shown in Fig. 1D, the wing-flapping frequency of bees in flight was analyzed for load masses of 5 to 60 mg.

### PEH design

**Fig. 2.**
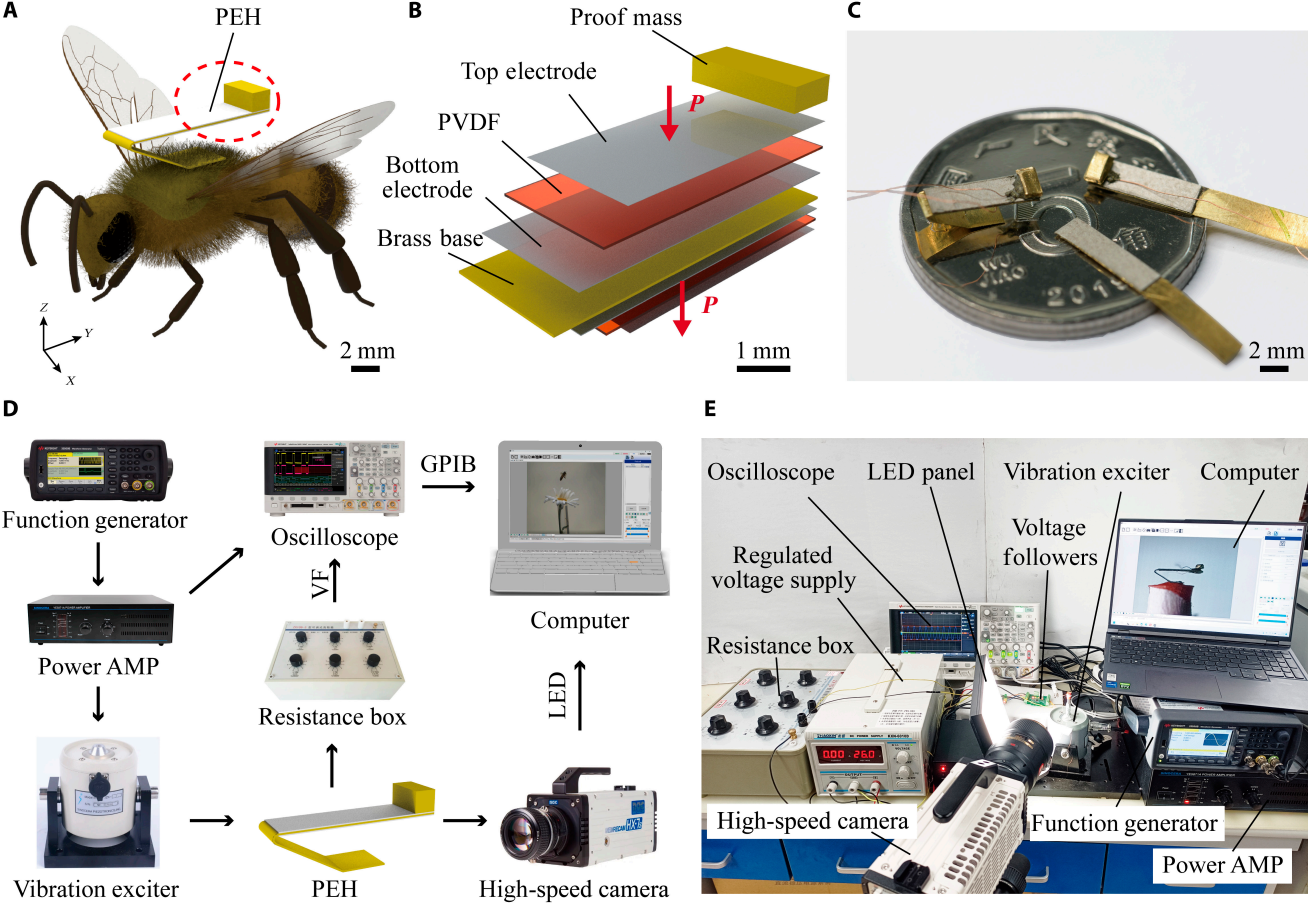
Design, fabrication, and test of the piezoelectric energy harvester (PEH). (A) Schematic diagram of the vibrational energy harvesting setup used in this study. (B) Schematic diagram of the exploded view of the PEH. (C) The PEH. (D) Schematic diagram and (E) photograph of the experimental setup used for evaluating the output performance of the PEH. PVDF, polyvinylidene fluoride; AMP, amplifier; VF, voltage follower; GPIB, General-Purpose Interface Bus.

In general, a bee can carry more than half of its weight in flight [38]. However, when the bees’ load mass is not evenly distributed, it generates rotational inertia around the center of gravity, which affects the bees’ flight stability and maneuverability and also reduces the bee load capacity [39]. In this study, a piezoelectric beam structure that matches the center of gravity of the bees’ body is designed, as shown in Fig. 2A. The bees’ center of gravity is located approximately at the point where the anterior and posterior ventral segments join (Fig. S1). The designed parameters are adjusted so that the PEH and the bees’ center of gravity coincide. This reduces the inertial forces generated during the roll, pitch, and yaw processes, ensuring that their impact on the bees’ flight is minimized. We select polyvinylidene fluoride (PVDF) as a piezoelectric material to reduce the total mass of the PEH. The PEH adopts a double-crystal structure, which corresponds to the PEH layered structure shown in Fig. 2B in the red dashed-line circle in Fig. 2A. The PEH consists of a PVDF film (50 μm in thickness), silver electrodes at the top and bottom (5 μm in thickness), a copper substrate in the middle (80 μm in thickness), and a mass block on end. The red arrows in Fig. 2B indicate the polarization direction of PVDF films, which are all downward perpendicular to the thickness since parallel connections are used in this research. The strain direction of PVDF is vertical to the polarization direction (along the *Y* axis); thus, the operating mode of the PEH is *d*_31_.

### PEH fabrication and testing methods

In this study, a commercial PVDF film (San San Intelligent Technology, China) was used directly, and the properties of materials are shown in the Table. Then, the PVDF film was bonded to the copper substrate surface (obtained by laser cutting) using conductive adhesive (Fig. S2). To improve the bonding strength, the surface of the copper substrate was properly sanded before bonding. A 50-μm enameled wire was bonded to the ends of the top and bottom PVDF film surfaces, and the mass block was bonded to the PEH end position. After bonding, the cross-section of the PEH was wiped with an alcohol swab to avoid a short circuit of the piezoelectric films. Next, the bonded PEH was placed in an oven at 50 °C for 2 h to allow the epoxy resin adhesive to solidify fully. Then, the PEH was removed from the oven, and an extrusion mold was used for the 3-dimensional structure of the PEH; namely, the mold was created by 3-dimensional printing. Finally, the PEH was tested using a multimeter to ensure that no short circuit occurred, and a layer of insulating paint was sprayed on the PEH surface (Fig. 2C).

**Table.** Material properties of the PVDF film

Parameters	Symbol	Value
Density	*ρ*	7,900 kg/m^3^
Piezoelectric constant	*d*_31_	32 pC/N
	*d*_33_	−28 pC/N
Young’s modulus	*E*	2.8 GPa
Relative permittivity	*ε*_r_	13.5
Electromechanical coupling factor	*k*	13%

We tested the PEH’s output using a vibration experimental system. The experimental system used to study the PEH’s output performance is presented in Fig. 2D. The experimental setup included a PEH, a signal generator (33510B, Yestech, USA), a power amplifier (YE5871A, Unitech, China), and an electromagnetic exciter (JZK-2, Unitech, China). A digital oscilloscope (DSOX3034T, Yestech, USA) was used to measure the output voltage across the load resistor and display the power amplifier’s output signal to determine the output parameters of the shaker. The voltage following module was used to isolate the effect of high resistance on the output voltage. A high-speed CMOS camera (Memrecam HX-7S, NAC, Japan) was used to record the PEH vibration data for subsequent end displacement analysis of the PEH. A light-emitting diode panel light was used to fill in the light. A computer was used to control the high-speed CMOS camera and digital oscilloscope and store the collected data. The photograph of the experimental setup is shown in Fig. 2E.

The proposed PEH device was bonded to the bees’ thorax using an adhesive; the experimental process referred to the bonding of mass blocks, as explained in the “Bees’ wing-flapping frequency tests” section. When the bee regained its activity, it was made to flip over on the ground to test its ability to self-stand after the flip. In addition, the bee-carrying PEH was allowed to fly in the experimental screening. Then, the bee was allowed to fly around the room and its flight behavior was observed. In this experiment, the bees were taken out of the hive randomly. To avoid individual differences, we tested a total of 5 bees.

## Results and Discussion

### Wing-flapping frequency of bees

**Fig. 3.**
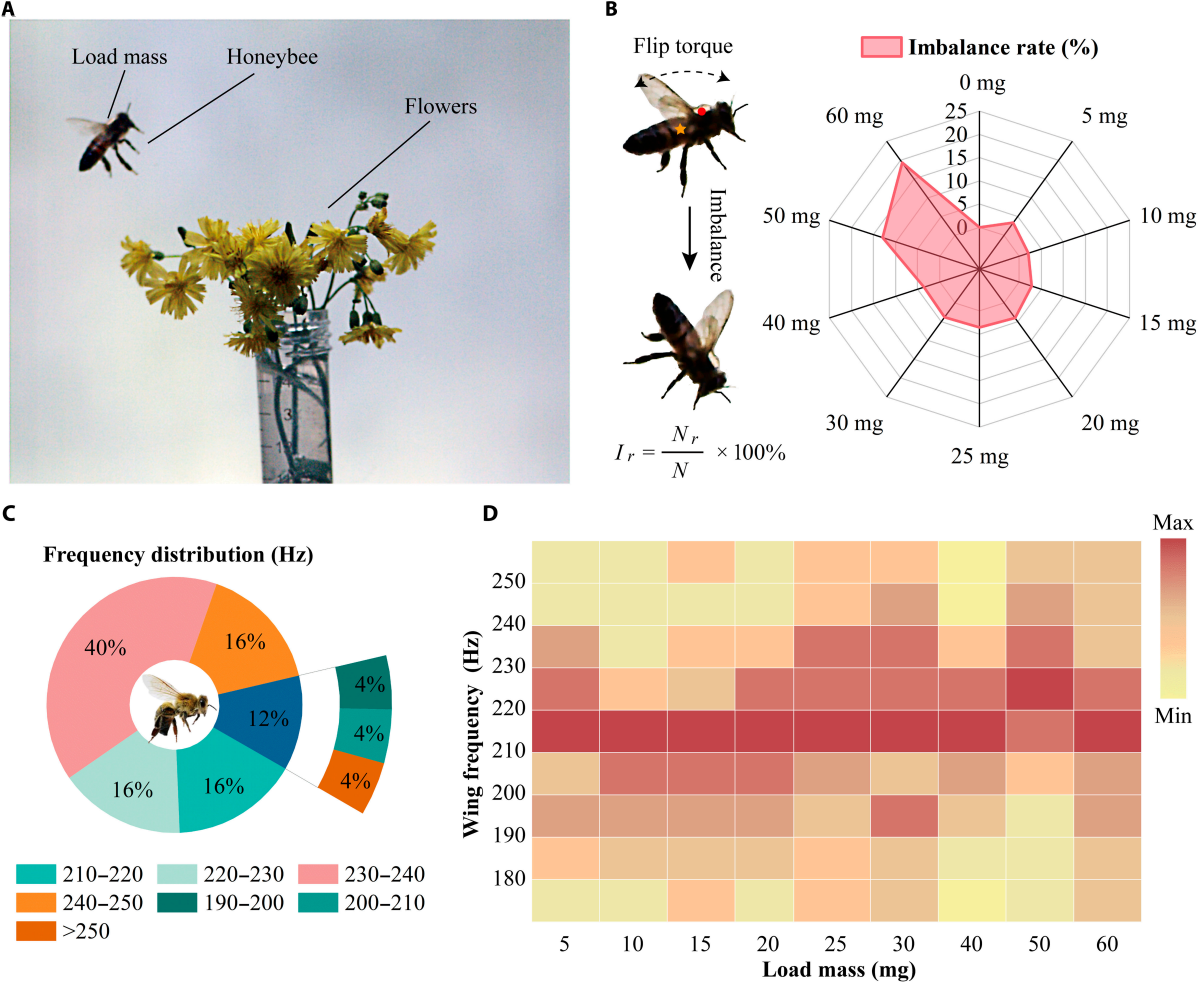
A bee’s flights under different load conditions. (A) A bee flying under the load mass was photographed by a high-speed CMOS camera. (B) Imbalance rate ($I_{r}$) under different load masses. (C) The frequency distribution of the bee wing flapping with no load mass. (D) The concentrated frequency interval of bees under different load masses.

The flight of a loaded bee recorded by the high-speed camera during the experiment is presented in Fig. 3A, and the corresponding flight movie is given in Video S1. In this experiment, 5 bees were tested separately under the same load mass, and each bee was recorded for at least 5 flights. The observational study showed that the flight ability of bees decreased gradually with the increase in load mass. The number of bee flight imbalances at different load masses was counted separately, and the imbalance rate was used to describe the effect of the load mass on the bee’s flight behavior. The rate was calculated as follows:

$$I_{r}=\frac{N_{r}}{N}\times 100\%$$ (1)

where *N* is the number of experiments at a specific load mass and $N_{r}$ is the number of bee flight imbalances.

The imbalance of the bee flight was generated by the inertia of rotation in the pitch direction caused by the gravity center of load mass not being coincident with the bees, as shown in Fig. 3B. According to the experimental results, the flight imbalance rate remained relatively stable at approximately 3% under the load mass from 0 to 40 mg. When the weighted mass reached 50 mg, the imbalance rate suddenly increased to 12.9%; at a 60-mg load mass, the imbalance rate reached 19.4%. These results were consistent with the observation results; namely, the flight ability of bees decreased with the load mass. These results indicate the necessity of considering the basic balance of the device’s mass and center of gravity when designing a PEH for honeybee vibration energy harvesting.

A statistical analysis of the bee wing-flapping concentration frequency band was conducted to match the PEH resonant frequency better with the bee thorax vibration. Shown in Fig. 3C at no load mass, the frequency of the bee’s wing flapping was concentrated between 230 and 240 Hz. These results were consistent with the findings of previous research [40]. However, under the load condition (5 to 60 mg), the frequency of the bees’ wings flapping was concentrated between 210 and 220 Hz, as shown in Fig. 3D. Thus, the wing-flapping frequency of the bee decreased after loading the mass in the thoracic region. The specific percentages of the bee wing-flapping frequency intervals for different load masses are shown in Fig. S3. According to Jankauski’s conclusion [37], the vibration frequency of the bee’s thorax is equal to the wing-flapping frequency, so the data obtained from this experiment could provide a very effective reference for the PEH design intended for bees.

### PEH performances

**Fig. 4.**
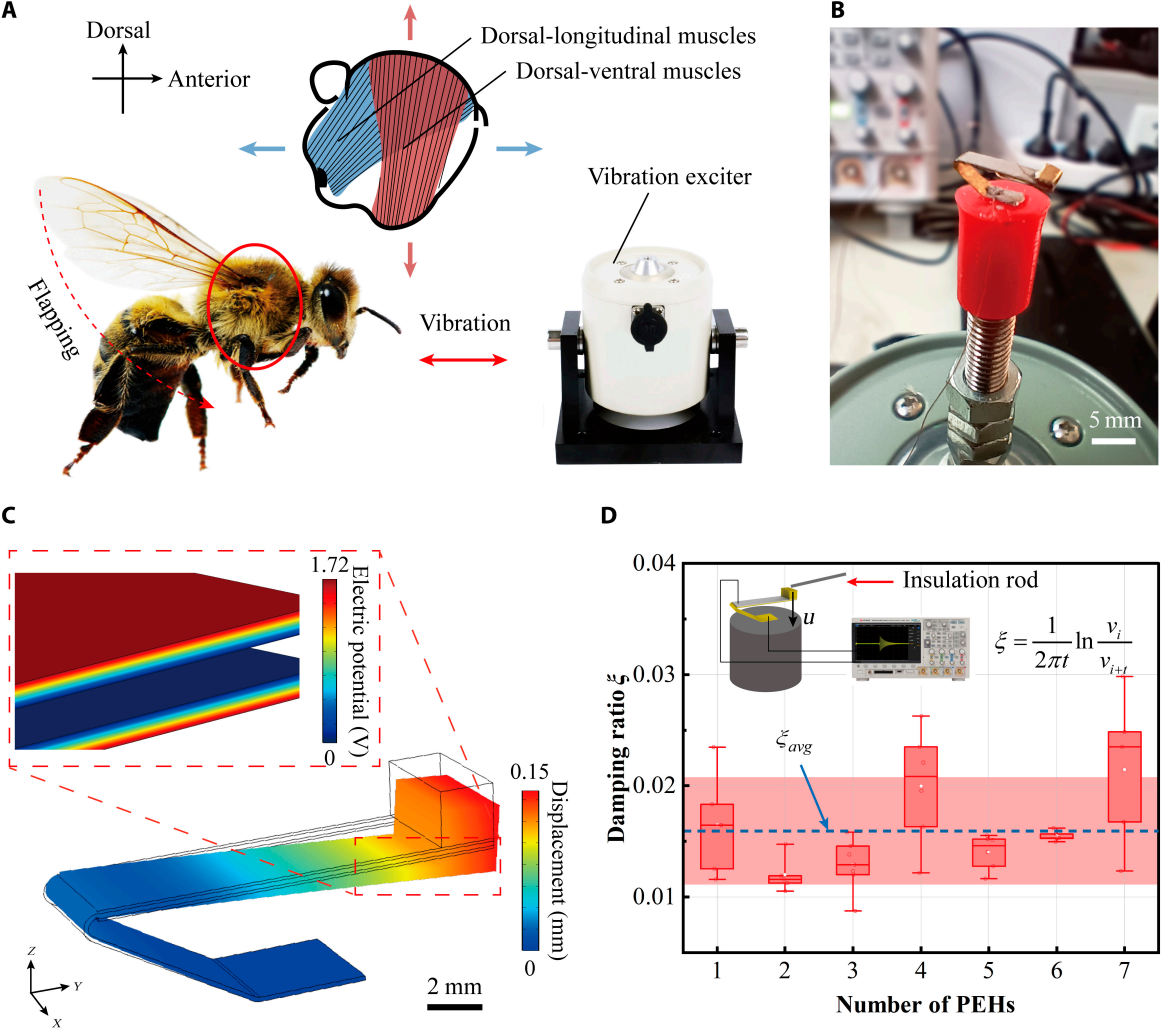
The vibration characteristics and electrical output results of the PEH. (A) Schematic diagram of bee wing flapping and principle of the simulation experiment. (B) A photo of the PEH fixed on the exciter. (C) Distribution of PEH’s displacement and surface potential obtained by Comsol Multiphysics. (D) Damping test method and results of the PEH.

In this study, the PEH was used for vibration energy harvesting during the flight while being carried by bees, and it was difficult to study the energy conversion output using the bee body directly. However, the bee’s wing flapping is accomplished by contracting the dorsal-longitudinal and dorsal-ventral muscles [41], as shown in Fig. 4A. The proposed PEH is mainly intended for thoracic vertical vibration energy harvesting, so a vibration exciter was used to simulate the output of the bee’s thoracic vibration. A photograph of the PEH placed on the exciter in the experiment is presented in Fig. 4B.

As mentioned above, the end mass of the PEH was determined by means of a modal frequency-matching design. Considering that the basic parameters of the PEH were determined, the first-order modal frequency of the PEH was set in the range of 210 to 220 Hz based on the experimental results of the bee flight with the load mass. Finally, after simulation by Comsol Multiphysics, the modal frequency of the PEH was determined as 215 Hz. The simulation results of the displacement and potential distribution at the PEH resonance are presented in Fig. 4C; under 9.8 m/s^2^ (1 *g*), the maximum displacement and potential of the PEH were 0.15 mm and 1.72 V, respectively.

Next, the damping of the proposed PEH was tested, and the adopted test method is displayed in the inset of Fig. 4D. An insulating rod was used to introduce a displacement on the end of the PEH, and then the PEH was adapted to make a free decaying motion. An oscilloscope was used to record the output signal of the PEH. One of the output signals measured in the experiment is shown in Fig. S4. The damping of PEH was obtained by

$$\xi =\frac{1}{2\pi t}\ln\frac{v_{i}}{v_{i+t}}$$ (2)

where $v_{i}$ and $v_{i+t}$ denote the peak voltages and *t* is the interval between the 2 data points.

Seven PEHs were tested 5 times separately, and the results are shown in Fig. 4D, where it can be seen that the average reference range of PEH damping was between 0.012 and 0.021, and its average value was 0.016. Meanwhile, the damping coefficients in the Comsol model were defined by referring to the experimental results of PEH damping to make the simulation model more consistent with the actual situation.

**Fig. 5.**
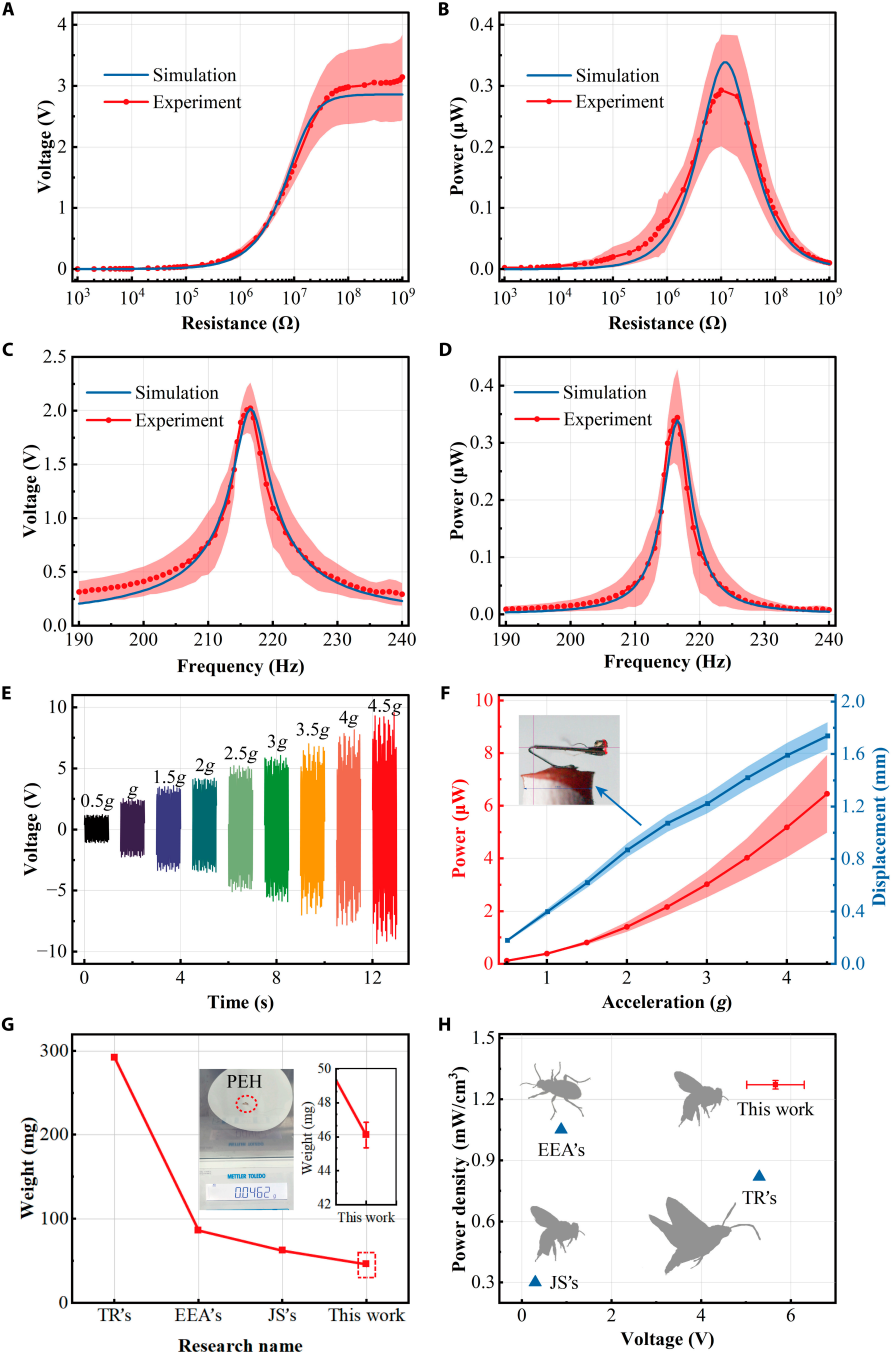
The PEH performance. (A and B) The PEH output at different resistances. (C and D) The PEH output at different frequencies. (E) The PEH voltage at different exciting acceleration values. (F) Power and displacement of the PEH at different exciting acceleration values. (G) Comparison of the proposed PEH with other insect vibration energy harvesters in terms of mass. The illustration is a photo of PEH weighing. (H) Comparison of the PEH’s output performance with the performances of other insect vibration energy harvesters. TR’s, Timothy Reissman’s; EEA, Ethem Erkan Aktakka’s; JS’s, Jake Shearwood’s.

The basic output of the PEH was measured under excitation at 9.8 m/s^2^ (1 *g*). We conducted resistance and frequency response tests on the 4 PEHs prepared separately. At the first modal frequency, the PEH output was tested as a function of resistance. The test results indicated that the maximum load voltage of the PEH reached more than 3 V, as shown in Fig. 5A. In addition, the PEH could convert the maximum average power to 0.3 μW under a load resistance of 10 MΩ (Fig. 5B). After connecting a 10-MΩ load resistance into the circuit, the PEH output was tested in the frequency range from 190 to 240 Hz, as shown in Fig. 5C and D. 45 We can observe that the load voltage at the resonant frequency was up to 2 V, and the power reached 0.3 μW. In addition, the PEH had an effective output in the vibration frequency ranging from 210 to 220 Hz. In Fig. 5A to D, the simulation and experimental results coincided well, which proved the correctness of the constructed simulation model. The output display during PEH vibration tests is given in Video S2.

According to the study by Pritchard and Vallejo-Marín [42], the acceleration in the thoracic of honeybees is generally high, and its maximum can reach a value of 49.3 m/s^2^. Based on this conclusion, the PEH output was studied under different acceleration vibration excitation values. The output voltage under acceleration from 0.5 *g* to 4.5 *g* (*g* = 9.8 m/s^2^) is presented in Fig. 5E, in which the maximum output voltage of the PEH was up to 8 V. The output power and the end vibration displacement of the proposed PEH at different accelerations are presented in Fig. 5F, where it can be seen that the PEH power increased nonlinearly with the acceleration, and the maximum output power reached 6.5 μW. In addition, the displacement curve result indicated that the maximum displacement at the PEH end was 1.75 mm, which was lower than the height of the proposed PEH. This further means that when the bee is flying with the proposed PEH, even if the thorax is in the maximum vibration state, it will not touch the end of the PEH with the bee’s body directly. As mentioned above, the displacement of the PEH was measured by a high-speed CMOS camera. The analysis image of the Tracker software is presented in Fig. 5F.

Compared with other PEHs used for insect vibration energy harvesting, namely, Ethem Erkan Aktakka’s PEH for beetles [20], Jake Shearwood’s PEH for bees [43], and Timothy Reissman’s PEH for moths [44], the mass of the proposed PEH is smaller, having a value of only 46 mg, as shown in Fig. 5G. In addition, as shown in Fig. 5H, the proposed PEH achieved better results than the other PEHs in terms of output performance. The maximum average output voltage and power density of the proposed PEH were 5.66 V and 1.27 mW/cm^3^, respectively. This result was much better than those presented in related studies [20,43,44]. In particular, compared to the same PEH (Jake Shearwood’s) used for honeybee vibration energy harvesting, our PEH could improve the voltage and power density by a level.

### Flight performance of the PEH carried by an insect

To illustrate that the bees were able to carry the PEH in real-life situations, the PEH was fixed on top of a bee’s thorax, as shown in Fig. S5(a). The bee’s ability to right itself after tipping over while carrying the PEH was examined, and the results indicate that the bees could right their bodies within 2 s when carrying the PEH, as shown in Fig. S5(b) and Video S3. The bees were placed inside the screen, and their flight behavior was observed. The results indicated that the bees could normally fly on the screen, and their flight ability did not decrease appreciably (Video S3).

Finally, the screen seal was released so that the bees could fly out of the screen directly. The results showed that the bees were flying out and then went directly toward the lights in the room, hovering around the light at last. This state was not appreciably different from the data observed in unloaded bees in practice. The results of this study indicate that bees can normally fly with the proposed PEH, which proves the effectiveness of the proposed design scheme.

## Conclusion

In this study, we established a design strategy to attach a PEH on bees through systematic evaluation of motion abilities and demonstrated the bees freely flying while carrying the PEH. During our investigation, we found changes in the flight imbalance rate and frequency concentration interval of bees when carrying different load masses. Combining the bee’s load mass and center of gravity distribution, a PEH balanced with the bees is designed based on the resonant-frequency-matching method. The PEH is verified by simulation and actual tests, and the results show that our PEH has a small mass and superior energy output performance. These outcomes confirm the efficacy of our PEH in practical applications. Furthermore, we investigated the impact of the PEH on the basic behavior of bees. The results revealed that when carrying the PEH, the fundamental behavior of bees remained largely unchanged. Notably, the bees exhibited the ability to fly freely and normally with the proposed PEH, indicating that the device’s integration did not disrupt their natural flying capabilities.

Compared to the existing solutions [33], our PEH stands out with its lower mass while boasting superior performance in terms of output voltage and power density. Moreover, through effective management and storage of the converted energy, the PEH exhibits the potential to function as a semipermanent power source. This remarkable capability makes it well suited for providing energy to low-power electronics (microwatt power consumption), such as wireless sensors and controllers carried by ICBs. The approach presented in this study not only benefits ICBs but also holds promise for other flying insects. By achieving continuous energy conversion and supply, it extends the one-time service life of ICBs and expands their range of activity. This advancement has the potential to revolutionize the field of energy supply for ICBs, enabling them to sustain their operations for extended periods.

## Data Availability

The data used to support the findings of this study are available from the corresponding authors upon request.

## Supplementary Materials

Supplementary 1 Figs. S1 to S5 Videos S1 to S3

## References

[1] Ando N, Kanzaki R. Insect-machine hybrid robot: Closing loops with mobile robots. Curr Opin Insect Sci. 2020;42:61–69. 32992040 10.1016/j.cois.2020.09.006

[2] Goyal P, Cribellier A, de Croon GC, Lankheet MJ, van Leeuwen JL, Pieters RP, Muijres FT. Bumblebees land rapidly and robustly using a sophisticated modular flight control strategy. iScience. 2021;24(5): Article 102407. 33997689 10.1016/j.isci.2021.102407 PMC8099750

[3] Rasakatla S, Tenma W, Suzuki T, Indurkhya B, Mizuuchi I. CameraRoach: A WiFi-and camera-enabled cyborg cockroach for search and rescue. J Robot Mechatron. 2022;34(1):149–158.

[4] Shoji K, Morishima K, Akiyama Y, Nakamura N, Ohno H. Autonomous environmental monitoring by self-powered biohybrid robot. In: *2016 IEEE international conference on mechatronics and automation*. New York (NY): IEEE; 2016. p. 629–634.

[5] Yu Y, Wu Z, Xu K, Gong Y, Zheng N, Zheng X, Pan G. Automatic training of rat cyborgs for navigation. Comput Intell Neurosci. 2016;2016: Article 6459251. 27436999 10.1155/2016/6459251 PMC4942600

[6] Dirafzoon A, Bozkurt A, Lobaton E. A framework for mapping with biobotic insect networks: From local to global maps. Robot Auton Syst. 2017;88:79–96.

[7] Boukoberine MN, Zhou Z, Benbouzid M. A critical review on unmanned aerial vehicles power supply and energy management: Solutions, strategies, and prospects. Appl Energy. 2019;255: Article 113823.

[8] Wei F, Yin C, Zheng J, Zhan Z, Yao L. Rise of cyborg microrobot: Different story for different configuration. IET Nanobiotechnol. 2019;13(7):651–664. 31573533 10.1049/iet-nbt.2018.5374 PMC8676360

[9] Li Y, Wu J, Sato H. Feedback control-based navigation of a flying insect-machine hybrid robot. Soft Robot. 2018;5(4):365–374. 29722607 10.1089/soro.2017.0118

[10] Yu L, Zhao J, Ma Z, Wang W, Yan S, Jin Y, Fang Y. Experimental verification on steering flight of honeybee by electrical stimulation. Cyborg Bionic Syst. 2022;2022: Article 9895837. 39886318 10.34133/2022/9895837 PMC11780726

[11] Ma S, Liu P, Liu S, Li Y, Li B. Launching of a cyborg locust via co-contraction control of hindleg muscles. IEEE Trans Robot. 2022;38(4):2208–2219.

[12] Fu F, Li Y, Wang H, Li B, Sato H. The function of pitching in beetle’s flight revealed by insect-wearable backpack. Biosens Bioelectron. 2022;198: Article 113818. 34861525 10.1016/j.bios.2021.113818

[13] Liu P, Ma S, Liu S, Li Y, Li B. Omnidirectional jump control of a locust-computer hybrid robot. Soft Robot. 2022;10(1):40–51. 35333662 10.1089/soro.2021.0137

[14] Schwefel J, Ritzmann RE, Lee IN, Pollack A, Weeman W, Garverick S, Willis M, Rasmussen M, Scherson D. Wireless communication by an autonomous self-powered cyborg insect. J Electrochem Soc. 2014;161: Article H3113.

[15] Ben Amar A, Kouki AB, Cao H. Power approaches for implantable medical devices. Sensors. 2015;15:28889–28914. 26580626 10.3390/s151128889 PMC4701313

[16] Ma Z, Zhao J, Yu L, Yan M, Liang L, Wu X, Xu M, Wang W, Yan S. A review of energy supply for biomachine hybrid robots. Cyborg Bionic Syst. 2023;4: Article 0053. 37766796 10.34133/cbsystems.0053 PMC10521967

[17] Pan M, Yuan C, Liang X, Zou J, Zhang Y, Bowen C. Triboelectric and piezoelectric nanogenerators for future soft robots and machines. iScience. 2020;23(11): Article 101682. 33163937 10.1016/j.isci.2020.101682 PMC7607424

[18] Ntawuzumunsi E, Kumaran S, Sibomana L. Self-powered smart beehive monitoring and control system (SBMaCS). Sensors. 2021;21(10): Article 3522. 34069366 10.3390/s21103522 PMC8158743

[19] Zhang H, Wu X, Pan Y, Azam A, Zhang Z. A novel vibration energy harvester based on eccentric semicircular rotor for self-powered applications in wildlife monitoring. Energy Convers Manag. 2021;247: Article 114674.

[20] Aktakka EE, Kim H, Najafi K. Energy scavenging from insect flight. J Micromech Microeng. 2011;21(9): Article 095016.

[21] Li H, Tian C, Lu J, Myjak MJ, Martinez JJ, Brown RS, Deng ZD. An energy harvesting underwater acoustic transmitter for aquatic animals. Sci Rep. 2016;6: Article 33804. 27647426 10.1038/srep33804 PMC5029286

[22] Sargolzaeiaval Y, Ramesh VP, Neumann TV, Misra V, Vashaee D, Dickey MD, Öztürk MC. Flexible thermoelectric generators for body heat harvesting—Enhanced device performance using high thermal conductivity elastomer encapsulation on liquid metal interconnects. Appl Energy. 2020;262: Article 114370.

[23] Zhang S, Zhou Y, Liu Y, Wallace GG, Beirne S, Chen J. All-polymer wearable thermoelectrochemical cells harvesting body heat. iScience. 2021;24: Article 103466. 34927022 10.1016/j.isci.2021.103466 PMC8649731

[24] Siddique ARM, Mahmud S, Van Heyst B. A review of the state of the science on wearable thermoelectric power generators (TEGs) and their existing challenges. Renew Sust Energ Rev. 2017;73:730–744.

[25] Shoji K, Akiyama Y, Suzuki M, Nakamura N, Ohno H, Morishima K. Biofuel cell backpacked insect and its application to wireless sensing. Biosens Bioelectron. 2016;78:390–395. 26655178 10.1016/j.bios.2015.11.077

[26] Nasar A, Perveen R. Applications of enzymatic biofuel cells in bioelectronic devices—A review. Int J Hydrog Energy. 2019;44(29):15287–15312.

[27] Lee D, Jeong SH, Yun S, Kim S, Sung J, Seo J, Son S, Kim JT, Susanti L, Jeong Y, et al. Totally implantable enzymatic biofuel cell and brain stimulator operating in bird through wireless communication. Biosens Bioelectron. 2021;171: Article 112746. 33113388 10.1016/j.bios.2020.112746

[28] Kakei Y, Katayama S, Lee S, Takakuwa M, Furusawa K, Umezu S, Sato H, Fukuda K, Someya T. Integration of body-mounted ultrasoft organic solar cell on cyborg insects with intact mobility. npj Flex Electron. 2022;6(1): Article 78.

[29] Song K, Han JH, Lim T, Kim N, Shin S, Kim J, Choo H, Jeong S, Kim YC, Wang ZL, et al. Subdermal flexible solar cell arrays for powering medical electronic implants. Adv Healthc Mater. 2016;5(13):1572–1580. 27139339 10.1002/adhm.201600222

[30] Bozkurt A, Lobaton E, Sichitiu M. A biobotic distributed sensor network for under-rubble search and rescue. Computer. 2016;49(5):38–46.

[31] Safaei M, Sodano HA, Anton SR. A review of energy harvesting using piezoelectric materials: State-of-the-art a decade later (2008–2018). Smart Mater Struct. 2019;28: Article 113001.

[32] Chang SC. A 1-mW vibration energy harvesting system for moth flight-control applications [thesis]. [Cambridge (MA)]: Massachusetts Institute of Technology; 2010.

[33] Shearwood J, Aldabashi N, Eltokhy A, Franklin EL, Raine NE, Zhang C, Palmer E, Cross P, Palego C. *C*-band telemetry of insect pollinators using a miniature transmitter and a self-piloted drone. IEEE Trans Microw Theory Techniques. 2021;69(1):938–946.

[34] Altshuler DL, Dickson WB, Vance JT, Roberts SP, Dickinson MH. Short-amplitude high-frequency wing strokes determine the aerodynamics of honeybee flight. Proc Natl Acad Sci USA. 2005;105(50):18213–18218. 10.1073/pnas.0506590102 PMC1312389 16330767

[35] Vallejo-Marin M. How and why do bees buzz? Implications for buzz pollination. J Exp Bot. 2022;73(4):1080–1092. 34537837 10.1093/jxb/erab428 PMC8866655

[36] Snodgrass RE, Eickwort GC. *Principles of insect morphology*. Ithaca (NY): Cornell University Press; 2018.

[37] Jankauski MA. Measuring the frequency response of the honeybee thorax. Bioinspir Biomim. 2020;15(4): Article 046002. 32209745 10.1088/1748-3190/ab835b

[38] Mountcastle AM, Ravi S, Combes SA. Nectar vs. pollen loading affects the tradeoff between flight stability and maneuverability in bumblebees. Proc Natl Acad Sci USA. 2015;112(33):10527–10532. 26240364 10.1073/pnas.1506126112 PMC4547240

[39] Polidori C, Federici M, Trombino L, Barberini V, Barbieri V, Andrietti F. Weight, volume and unbalancing: Loading constraints of mud dauber wasps carrying mud balls. J Zool. 2009;279:187–194.

[40] Sotavalta O. Flight-tone and wing-stroke frequency of insects and the dynamics of insect flight. Nature. 1952;170(4338):1057–1058. 13013315 10.1038/1701057a0

[41] Deora T, Gundiah N, Sane SP. Mechanics of the thorax in flies. J Exp Biol. 2017;220(8):1382–1395. 28424311 10.1242/jeb.128363

[42] Pritchard DJ, Vallejo-Marín M. Floral vibrations by buzz-pollinating bees achieve higher frequency, velocity and acceleration than flight and defence vibrations. J Exp Biol. 2020;223(11): Article jeb220541. 32366691 10.1242/jeb.220541

[43] Shearwood J, Hung D, Palego C, Cross P. Energy harvesting devices for honey bee health monitoring. In: *2017 IEEE MTT-S international microwave workshop series on advanced materials and processes for RF and THz applications (IMWS-AMP)*. New York (NY): IEEE; 2017. pp. 1–3.

[44] Ghasemi-Nejhad MN, Reissman T, MacCurdy RB, Garcia E. Electrical power generation from insect flight. Paper presented at: SPIE Smart Structures and Materials + Nondestructive Evaluation and Health Monitoring. Active and Passive Smart Structures and Integrated Systems; 2011 Apr 26; San Diego, CA.

