# Supplementary material for: Piezoelectric Energy Harvesting from the Thorax Vibration of Freely Flying Bees
Source: Cyborg Bionic Syst. 2025 Feb 26;6:0210. doi: 10.34133/cbsystems.0210 (PMC11861424; doi:10.34133/cbsystems.0210)
Supplement: Supplementary 1 — Figs. S1 to S5 Videos S1 to S3 [file cbsystems.0210.f1.zip › cbsystems.0210.f1.docx]

**Piezoelectric energy harvesting from thorax vibration of** **freely flying bees**

**Zhiyun Ma^1^, Jieliang Zhao^1,^*, Li Yu^1^, Lulu Liang^1^, Zhong Liu^2^, Yongxia Gu^2^, Jianing Wu^3,^*, Wenzhong Wang^1,^*, Shaoze Yan^4^**

^1^ School of Mechanical Engineering, Beijing Institute of Technology, Beijing, 100081, P. R. China

^2^ School of Artificial Intelligence, Beijing Technology and Business University, Beijing, 100048, P. R. China.

^3^ Department of Advanced Manufacturing, Sun Yat-sen University, Shenzhen, 518107, P. R. China.

^4^ Department of Mechanical Engineering, Tsinghua University, Beijing, 100084, P. R. China.

* Corresponding author.

E-mail addresses: [jielzhao@126.com](mailto:jielzhao@126.com) (J. Zhao), [wujn27@mail.sysu.edu.cn](mailto:wujn27@mail.sysu.edu.cn) (J. Wu), [wangwzhong@bit.edu.cn](mailto:wangwzhong@bit.edu.cn) (W. Wang).

**Keywords:** energy harvesting; bees; thorax vibration; piezoelectric; MEMS; multi-physics field simulation

**Supplementary Text**

**1. Theoretical model for vibration-electricity conversion**

Generally, a vibration energy harvesting system can be modeled as a single-degree-of-freedom (SDOF) model, which was developed by Williams and Yates [1]. The SDOF modeling implies describing the dynamics of the free endpoints of a beam according to a set of lumped parameters, including the equivalent masses, stiffnesses, and damping of the beam, which are denoted by $\text{m}$, $\text{k}$, and $\text{d}_{\text{E}}$ respectively. Where $\text{d}_{\text{E}}$ consists of the mechanical damping factor $\text{d}_{\text{m}}$ and the electrically induced damping factor $\text{d}_{\text{e}}$. The SDOF model explains the conversion of mechanical vibrations to electrical energy, but it is valid only for collectors with linear damping and stiffness terms. In such systems, the mechanical damping and stiffness are proportional to the velocity and displacement, respectively.

External mechanical vibrations can be expressed by $\text{y}\left( \text{t} \right)\text{=}\text{Y}_{\text{0}}\text{sin(}\text{wt}\text{)}$*,* where $\text{Y}_{\text{0}}$ is the amplitude of the external excitation. Then, the differential equation of the system is given by:

$m\frac{d^{2}z(\mathrm{t})}{dt^{2}}+d_{E}\frac{dz(\mathrm{t})}{dt}+kz(t)=-m\frac{d^{2}y(\mathrm{t})}{dt^{2}}$ (S1)

where $\text{z}\left( \text{t} \right)$ and $\text{y}\left( \text{t} \right)$ denote spring deflection and input displacement, respectively.

Further, the relative motion of the equivalent masses can be obtained by:

 (S2)

where $\text{ω}$ and $\omega_{n}$ are the excitations and intrinsic frequencies of a spring-mass system, respectively.

The value of $\omega_{n}$ is calculated by:

$\omega_{n}=\sqrt{\frac{k}{m}}$ (S3)

and the phase angle $\text{φ}$ is obtained by:

 (S4)

Therefore, the energy dissipated within the damping can be calculated by[2]:

$P_{d}=\frac{m\zeta_{E}Y_{0}(\frac{w}{w_{n}})^{3}w^{3}}{[1-(\frac{w}{w_{n}})^{2}]^{2}+[2\zeta_{E}(\frac{w}{w_{n}})]^{2}}$ (S5)

where $\xi_{E}$ is the total damping ratio, and $\xi_{E}=d_{E}/2m\omega_{n}=\xi_{m}+\xi_{e}$.

The power converted to the electricity is equal to the power absorbed by the electrically induced damping $\text{d}_{\text{e}}$, and it is given by:

$P=\frac{m\zeta_{e}Y_{0}(\frac{w}{w_{n}})^{3}w^{3}}{[1-(\frac{w}{w_{n}})^{2}]^{2}+[2\zeta_{E}(\frac{w}{w_{n}})]^{2}}$ (S6)

When the excitation frequency $\text{ω}$ is equal to the intrinsic frequency $\text{ω}_{n}$, the spring-mass system can obtain the maximum energy, and in that case, the converted power is given by:

$P_{out}=\frac{m\zeta_{e}Y_{0}^{2}w_{n}^{3}}{4\zeta_{E}^{2}}$ (S7)

**2. Vibration-electricity conversion mechanisms**

Currently, there are three most popular and widely studied types of vibration-electricity conversion mechanisms: piezoelectric, electromagnetic, and electrostatic conversion [3]. Compared with the other two types of conversion mechanisms, the piezoelectric energy conversion mechanism dominates in terms of output voltage and power density Electromagnetic energy harvesting technology has a relatively low output voltage, so it requires multiple post-processing stages to meet the basic requirements of rechargeable voltage levels [4]. As for electrostatic energy harvesting methods, although their output voltage is generally high, they need to apply input voltage and current to make the capacitor unit vibrate [5].

In addition, with the development of thin film manufacturing technology, piezoelectric energy harvesting devices have been able to be fabricated on different scales, providing the advantages of simple basic structure, relatively lightweight, and easy application. The other approaches are greatly limited in terms of lightweight and small-scale applications due to the magnet, coil, and external input [6].

**References**

[1] C Williams, RB Yates. Analysis of a micro-electric generator for microsystems. sensors and actuators A: Physical. 1996;52(1-3):8-11. <http://dx.doi.org/10.1016/0924-4247(96)80118-x>

[2] SP Beeby, RN Torah, MJ Tudor, P Glynne-Jones, T O'Donnell, CR Saha, et al. A micro electromagnetic generator for vibration energy harvesting. Journal of Micromechanics and microengineering. 2007;17(7):1257. <http://dx.doi.org/10.1088/0960-1317/17/7/007>

[3] SP Beeby, MJ Tudor, N White. Energy harvesting vibration sources for microsystems applications. Measurement science and technology. 2006;17(12):R175. <http://dx.doi.org/10.1088/0957-0233/17/12/R01>

[4] A Erturk, DJ Inman. Piezoelectric energy harvesting: John Wiley & Sons, 2011.

[5] S Roundy, PK Wright, JM Rabaey. Energy scavenging for wireless sensor networks. Norwell: Springer; 2003. p. 45-7.

[6] KA Cook-Chennault, N Thambi, AM Sastry. Powering MEMS portable devices—a review of non-regenerative and regenerative power supply systems with special emphasis on piezoelectric energy harvesting systems. Smart materials and structures. 2008;17(4):043001. <http://dx.doi.org/10.1088/0964-1726/17/4/043001>


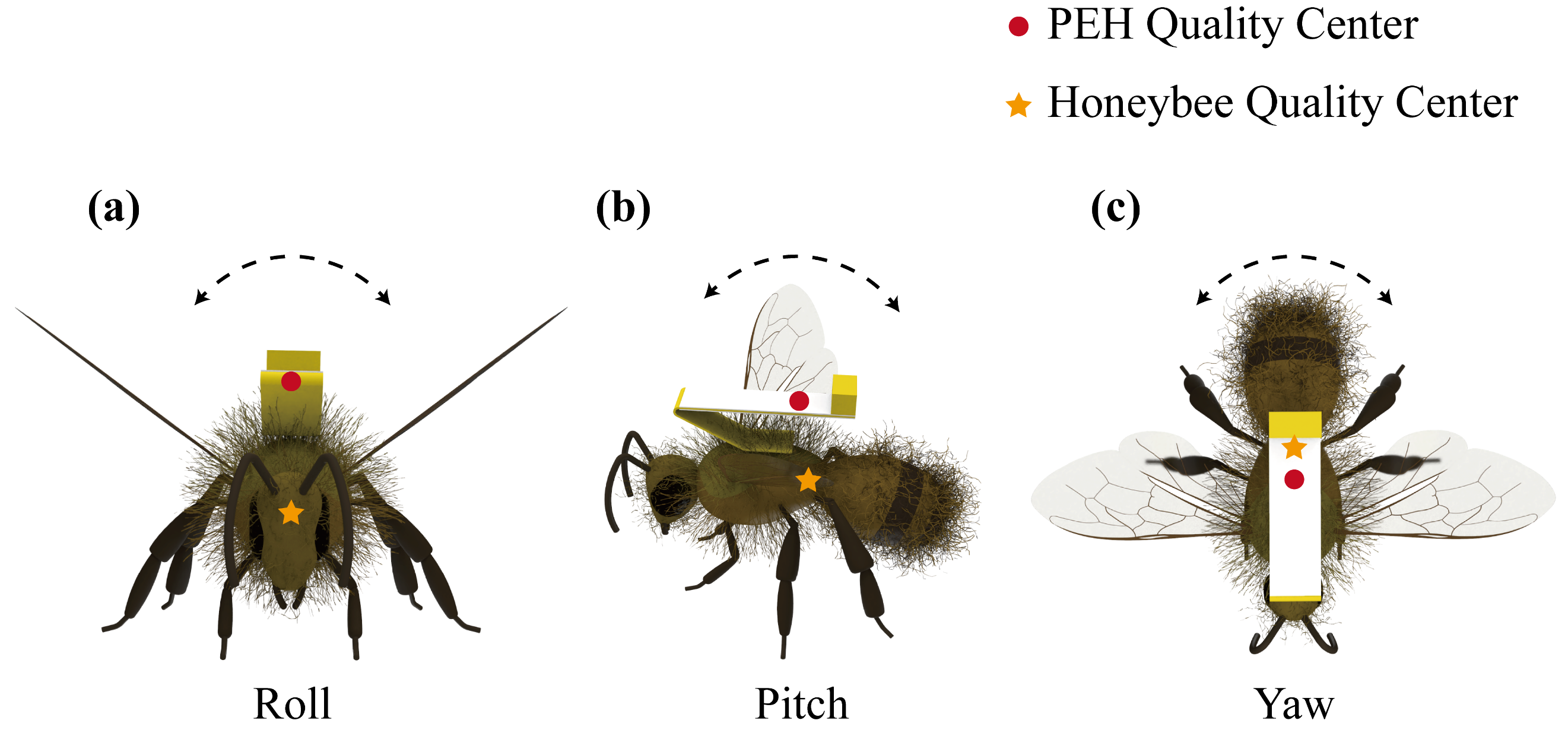


**Fig. S1.** Schematic representation of the honeybee and PEH Quality Center


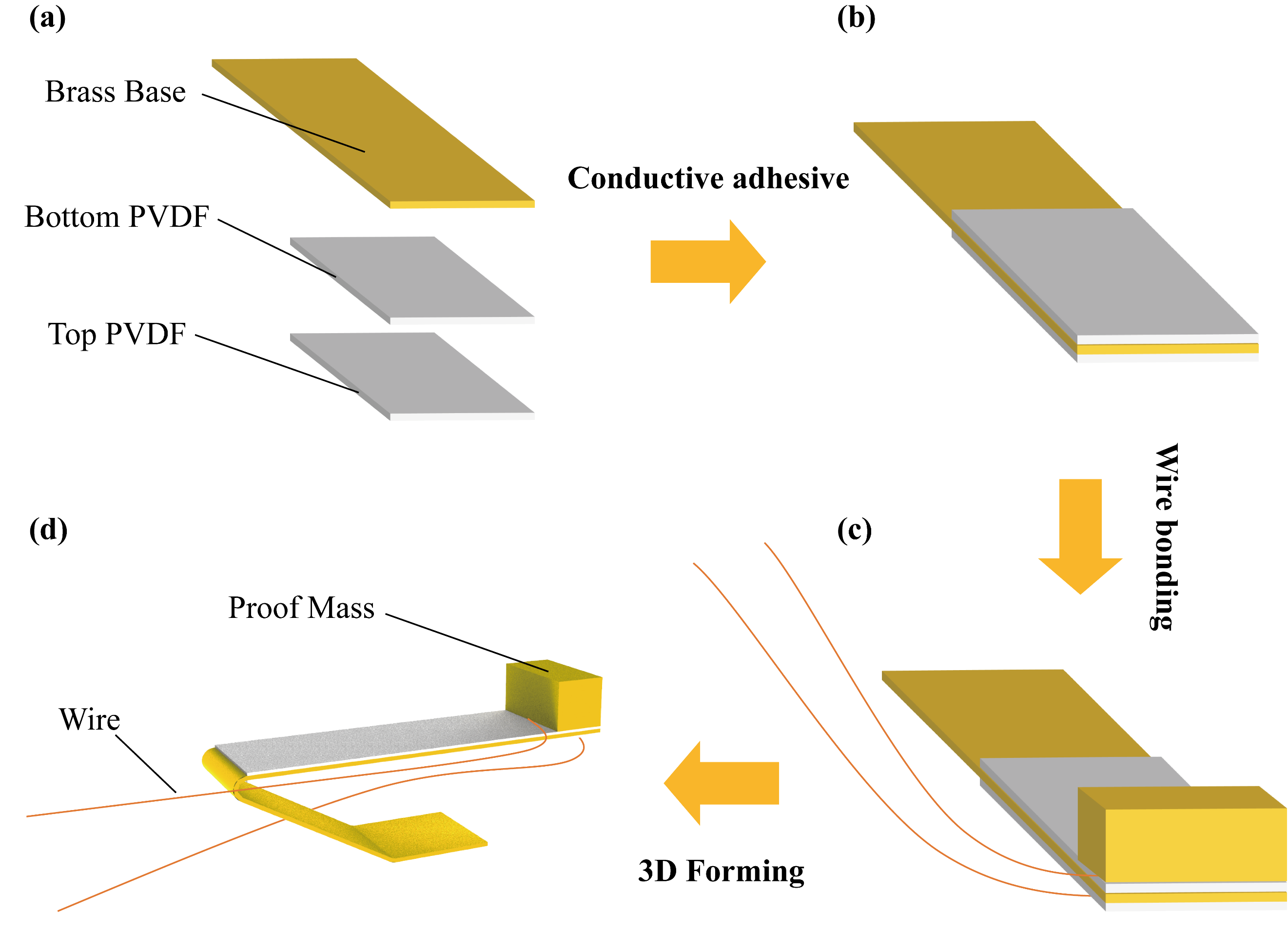


**Fig S2.** The fabrication process of PEH


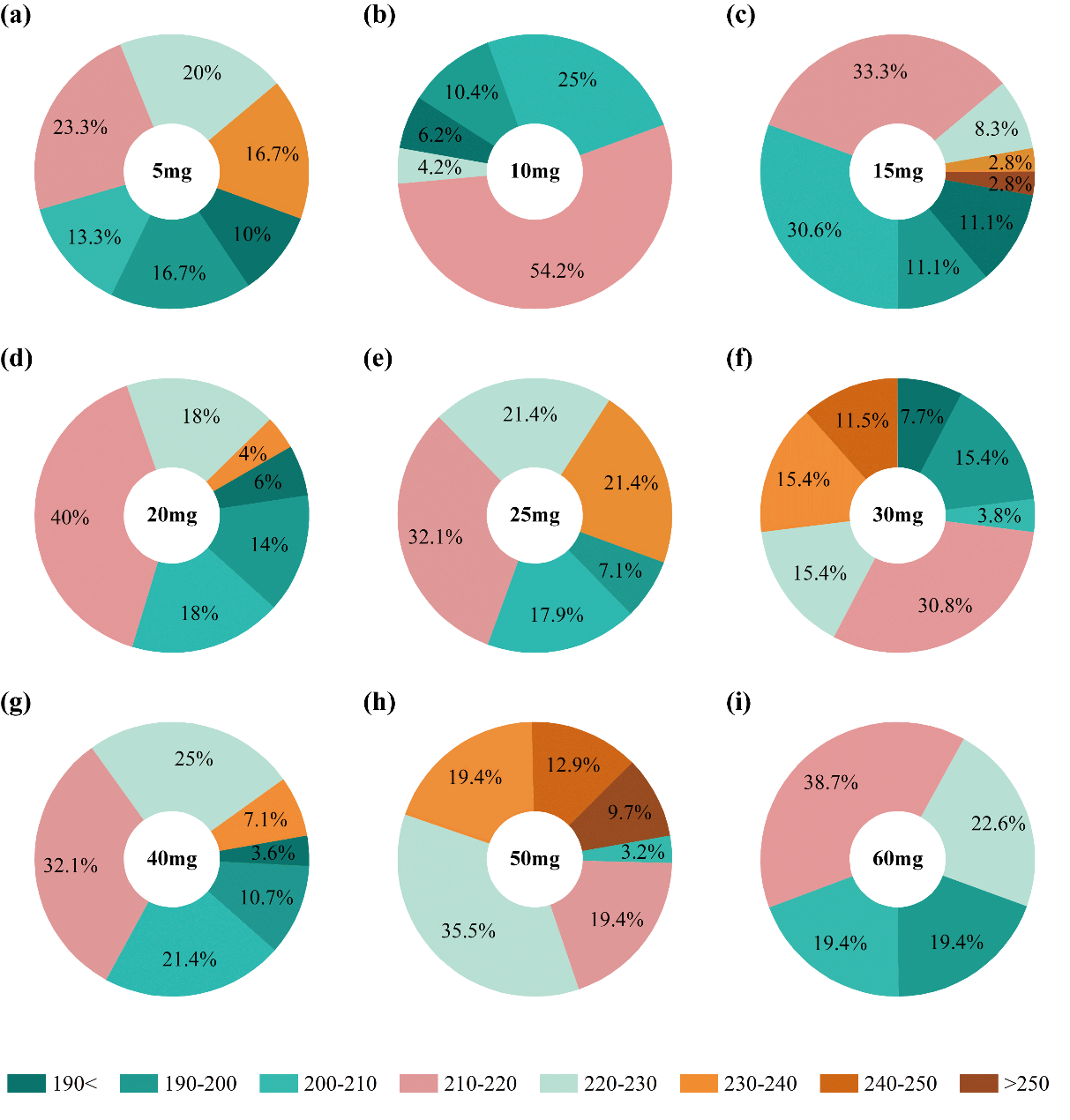


**Fig. S3.** Frequency distribution in different load mass


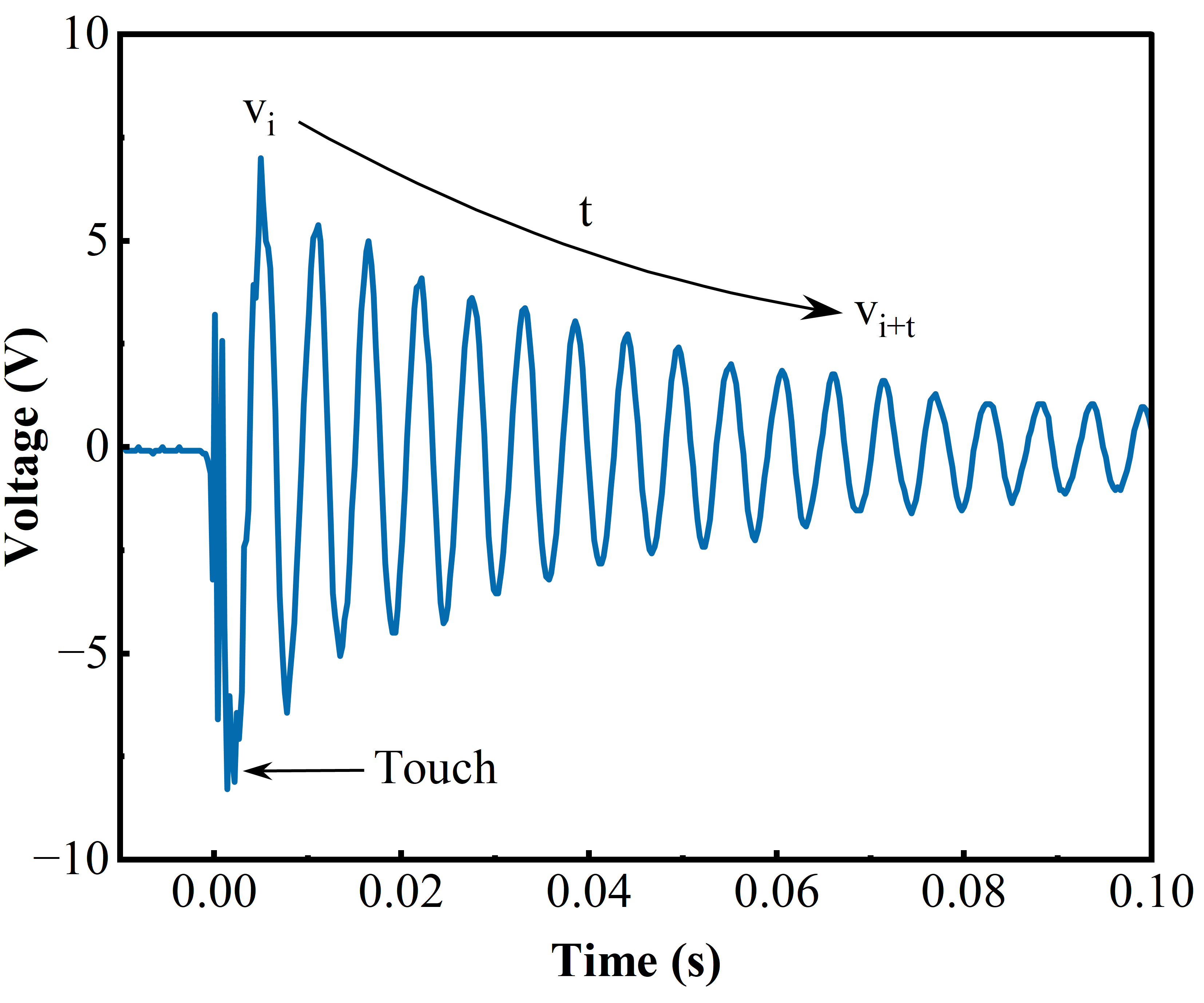


**Fig. S4.** Voltage decay signal of PEH after touching


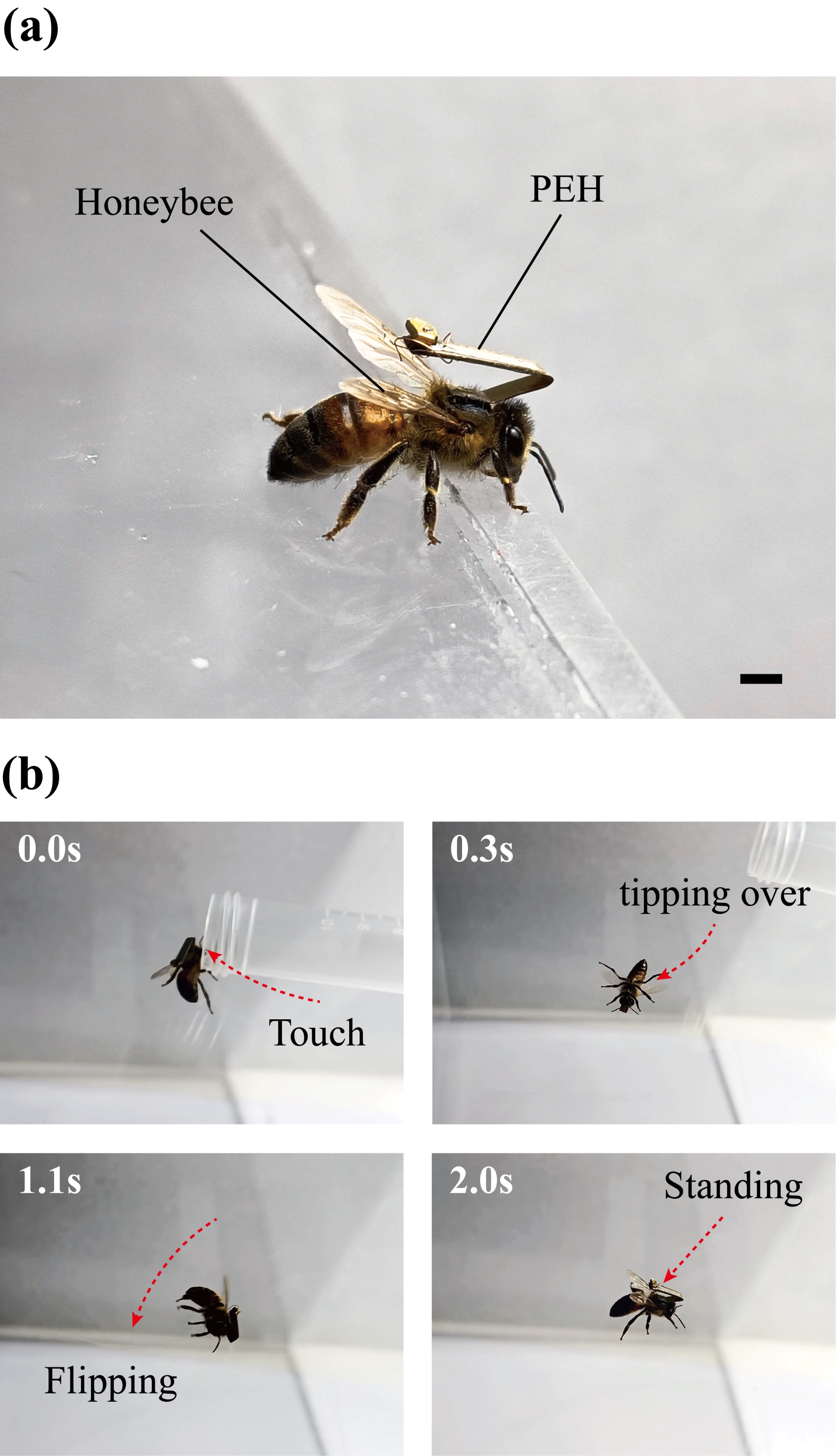


**Fig. S5.** Behaviour test of bees carrying PEH. (a) Photo of the bee carrying PEH. (b) The experiment on standing ability of bees after tipping over
